# National Estimates of hospital emergency department visits due to acute injuries associated with hookah smoking, United States, 2011–2019

**DOI:** 10.1186/s40621-020-00267-w

**Published:** 2020-08-10

**Authors:** Naa A. Inyang, Joanne T. Chang, Baoguang Wang

**Affiliations:** US Food and Drug Administration, Center for Tobacco Products, Office of Science, 10903 New Hampshire Avenue, Silver Spring, MD 20993-0002 USA

**Keywords:** Injuries, Hookah, Waterpipe, Emergency department visits, NEISS

## Abstract

**Introduction:**

Hookah (also known as waterpipe) smoking is associated with acute adverse health effects such as vomiting and fainting, symptoms related to carbon monoxide poisoning, and decreased pulmonary function, however, national estimates of hookah-related acute injuries are not currently available in the scientific literature. This study provides national estimates of United States hospital emergency department visits due to hookah-related acute injuries.

**Methods:**

We analyzed 2011–2019 data from the National Electronic Injury Surveillance System to calculate national estimates of emergency department visits due to hookah-related acute injuries. National Electronic Injury Surveillance System data were gathered from approximately 100 United States hospitals selected as a probability sample of approximately 5000 hospitals with emergency departments. Each case contains information abstracted from all emergency department records involving injuries associated with consumer products. All individuals admitted to emergency departments who sustained hookah-related acute injuries were included in the study.

**Results:**

During 2011–2019, an estimated 1371 (95% confidence interval: 505–2283) United States hospital emergency department visits were related to hookah-related acute injuries. The most common injuries were sustained from dizziness/light-headedness and syncopal episodes (54.8%), followed by burns (41.5%). Young adults aged 18–24 years accounted for 66.8% of hookah-related acute injuries admitted to United States emergency departments.

**Conclusions:**

This study provides national estimates of hospital emergency department visits due to hookah-related acute injuries. We found that hookah smoking related AIs mostly occurred among young adults. Study findings may inform public health policy and educational intervention efforts to prevent these events and complement other acute injury surveillance systems, such as the National Poison Data System.

## Introduction

Hookah (or waterpipe) smoking is a public health concern, particularly among adolescents and young adults. In 2019, 500,000 (3.4%) high-school students and 180,000 (1.6%) middle-school students in the United States (US) reported hookah smoking in the past 30 days (Wang et al. 2019). In 2013–2014, 10.7% of US young adults (aged 18–24 years) reported hookah smoking in the past 30 days (Kasza et al. 2017).

Hookah smoking has been associated with acute adverse health effects such as increased blood pressure and heart rate (Hakim et al. 2011; Kadhum et al. 2014; El-Zaatari et al. 2015), symptoms related to carbon monoxide (CO) poisoning (El-Zaatari et al. 2015; Retzky 2017), and long-term health effects such as decreased pulmonary function, chronic obstructive pulmonary disease (COPD), emphysema and asthma (Raad et al. 2011). In a typical session of hookah smoking (45 min), hookah users can inhale large amounts of toxic glycerol decomposition products (such as acrolein, acetaldehyde, and benzene), up to 12 times the amount of CO inhaled when smoking a single cigarette (Perraud et al. 2019), and as much smoke as 100 or more cigarettes (WHO Study Group on Tobacco Product Regulation (TobReg) n.d.).

A recent study using National Poison Data System (NPDS) data reported 276 hookah-related poisoning exposure calls to US poison control centers (PCCs) between 2005 and 2017, including events with reported symptoms that were consistent with CO poisoning (Rostron et al. 2019). However, NPDS data do not represent all poisoning events from hookah smoking at a national population level; they represent poisonings where individuals call a PCC. Nationally representative data on acute injuries related to hookah smoking is unknown. This study provides national estimates of hospital emergency department (ED) visits due to non-fatal acute injuries (AIs), including poisonings, related to hookah smoking using data from the National Electronic Injury Surveillance System (NEISS).

## Methods

### Data source

Data on hookah-related AIs admitted to EDs were obtained from NEISS, maintained by the US Consumer Product Safety Commission (CPSC). NEISS collects data involving consumer product-related emergency visits from approximately 100 US hospital EDs selected as a nationally-representative probability sample of the approximately 5000 US hospital EDs, with the goal of providing national estimates of the number of injuries admitted to EDs. For each case, information on demographics, location of incident, diagnosis, disposition and case narrative (including symptoms), was extracted from medical records and entered into the NEISS database by in-hospital NEISS case coordinators (US Consumer Product Safety Commission n.d.; US Consumer Product Safety Commission 2017a). This analysis focused on data from 2011 to 2019 to align with the years national surveys began collecting data on hookah smoking.

### Case extraction

We identified 53 cases of acute injuries related to hookah smoking presenting to US EDs in NEISS by conducting a text search using SAS (Version 9.4) within the case narrative text fields using the following keywords: “waterpipe,” “water_pipe,” “hookah,” “hooka,” “hukkah,” “hukka,” “shisha,” “boory,” “goza,” “narghile,” “nargile,” “arghile,” and “hubble.” All authors then independently reviewed all 53 case narratives for only hookah-related events and reconciled any discrepancies; we excluded 15 cases related to concurrent alcohol or illicit/unknown substance use (to isolate the effects of hookah smoking) and events that were unrelated to hookah smoking (i.e., cases where we could not clearly infer that the injuries were directly due to use of a hookah).

### Symptom coding

Two authors (JTC, BW) independently reviewed the resulting 38 case narratives and extracted symptoms known to be related to hookah smoking from the sample, such as, “burn,” “carbon monoxide poisoning,” “syncope,” “headache,” “nausea,” “dizziness,” “confusion,” “asthma,” “shortness of breath,” and other symptoms (El-Zaatari et al. 2015; Raad et al. 2011; Maarschalk et al. 2017). We then classified these symptoms into primary and secondary symptoms based on the nature of the incident. For example, for someone who felt dizzy after smoking hookah and then fell, the primary symptom was “dizziness” and the secondary symptom was “fall.”

### Statistical analysis

We calculated national estimates of hookah-related AIs admitted to US EDs by age group, sex, race, diagnosis, disposition, incident locale, and symptom. Due to the low number of cases for an analysis of single-year national estimate, we divided the nine-year study period into three three-year periods (2011–2013, 2014–2016, and 2017–2019 and calculated the national estimate. Analyses were conducted using SAS (Version 9.4) using the PROC SURVEY procedure to account for sample weights and complex sample design for hospitals.

## Results

From 2011 to 2019, 38 cases were identified in the NEISS data, representing a national estimate of 1371 (95% confidence interval (CI): 505–2238) hookah-related AIs admitted to US EDs (Table 1). The majority of cases occurred in individuals aged 18–24 years (66.8%). Over the nine-year study period, the national estimates were 99 (95% CI: 0, 246) for years 2011–2013; 518 (95% CI: 133, 902) for years 2014–2016, and 755 (95% CI: 145, 1365) for years 2017–2019. More than half (59.8%) of cases were female, and most cases occurred in whites (36.6%) or did not include information on race (30.4%).

**Table 1:** National Estimates of Acute Injuries Related to Hookah Smoking Admitted to US Emergency Departments in 2011–2019, by Age, Sex, and Race

	Unweighted N	National Estimate^**a**^	National Estimate or % (95% CI)
	38	1371	(505, 2238)
**Age (years)**			
0–5	2	73	5.3(0.0, 16.8)
6–11	0	–	–
12–17	3	142	10.3(0.0, 24.6)
18–24	23	916	66.8(45.7, 87.9)
25–34	7	154	11.3(4.4, 18.1)
35 and older	3	86	6.3(0.4, 12.2)
**Sex**			
Male	14	551	40.2(19.2, 61.1)
Female	24	821	59.8(38.9, 80.8)
**Race**			
White	11	502	36.6(23.7, 49.6)
Black	12	373	27.2(9.5, 44.9)
Other Race (including more than one race)	2	80	5.8(0.0, 14.3)
Not stated	13	416	30.4(10.5, 50.2)

^a^ National estimates were produced by applying statistical weights provided by US CPSC’s NEISS to the unweighted counts. CPSC considers an estimate to be unstable if (1) the estimate is less than 1200; (2) the number of records used is less than 20; or (3) the coefficient of variation is greater than 33% (US Consumer Product Safety Commission 2017b).

Table 2 shows the national estimate of hookah-related AIs by symptom, locale, diagnosis, and disposition. The most frequently documented symptom was burn (41.5%), followed by syncope (fainting) or falls (36.5%), dizziness or lightheadedness (18.3%), and other symptoms (3.6%), which included laceration, eye injury, and shortness of breath. Of the 17 burn cases, the narratives described two as having superficial burns and two as having second-degree/partial thickness burns; the remaining burn cases (*n* = 13) were unspecified. Most cases (46.1%) did not have information on incident locale; 38.3% occurred on public property and places of recreation or sports;15.6% of the cases occurred at home. The most common diagnoses were burns (thermal/scald) (41.5%), followed by internal organ injuries, concussion, or anoxia (34.3%); and contusions, lacerations, and avulsions (12.8%). The majority of cases (84.1%) were treated and released from the EDs, while 15.9% were treated and admitted, transferred, or kept for further observation.

**Table 2:** National Estimates of Acute Injuries Related to Hookah Smoking Admitted to US Emergency Departments in 2011–2019, by Symptom, Locale, Diagnosis, and Disposition

	Unweighted N	National Estimate^**a**^	National Estimate or % (95% CI)
	38	1371	(505, 2238)
**Symptom**^**b**^			
Burn	17	570	41.5(22.1, 61.0)
Dizziness/lightheadedness	5	252	18.3(1.2, 35.5)
Syncope/falls	13	500	36.5(15.7, 57.2)
Other^c^	3	50	3.6(0.0, 9.6)
**Incident Locale**^**d**^			
Home	6	214	15.6(1.9, 29.3)
Other public property^e^/Place of recreation or sports	13	525	38.3(12.9, 63.7)
Not recorded	19	633	46.1(26.5, 65.7)
**Diagnosis**			
Thermal burn/scald	17	570	41.5(22.1, 61.0)
Internal organ injury/concussion/anoxia	10	470	34.3(15.9, 52.6)
Contusions/lacerations/avulsion	6	176	12.8(1.7, 23.9)
Other/not stated^f^	5	156	11.4(0.0, 24.6)
**Disposition**			
Treated and released	33	1153	84.1(68.1, 100.0)
Treated and admitted, transferred, or kept for further observation^g^	5	218	15.9(0.0, 31.9)

^a^ National estimates were produced by applying statistical weights provided by US CPSC’s NEISS to the unweighted counts. CPSC considers an estimate to be unstable if (1) the estimate is less than 1200; (2) the number of records used is less than 20; or (3) the coefficient of variation is greater than 33% (US Consumer Product Safety Commission 2017b). Estimates may not add to N due to rounding

^b^ Symptom includes the primary symptoms coded from qualitative review of text narratives

^**c**^ Other symptoms include laceration, eye injury, and shortness of breath

^d^ Incident Locale is described by NEISS as “where the incident happened”

^e^ Other Public Property, as defined by NEISS, includes the following locations: Store, Office building, Restaurant, Church, Casino, Hotel or motel, Hospital/nursing home/other medical facility, Adult day care facility, Fraternity/Sorority house, Theater, Sidewalk (excl. Sidewalk of a house), Other public property, Parking lots/Parking garages

^f^ NEISS directs all case reporters to use diagnosis code 71 (defined as “Other/not stated”) when none of the other listed diagnoses are appropriate

^g^ Disposition “Treated and admitted, transferred, or and kept for further observation” includes cases whose dispositions were defined as treated and transferred to another hospital, treated and admitted for hospitalization (within same facility), and held for observation (includes admitted for observation)

## Discussion

We analyzed nationally representative data and estimated that there were 1371 ED visits due to hookah-related AIs in the US during 2011–2019. The estimate from this study complements findings (Rostron et al. 2019) from NPDS in understanding the overall burden of hookah-related AIs in the US. The estimated number of cases in this study is four times higher than that identified from NPDS during the similar period (Rostron et al. 2019), probably because some AI cases that needed immediate medical attention bypassed calling a PCC, and therefore were not captured in NPDS.

One of the most serious AIs of hookah smoking is acute CO poisoning. More than half of the cases in our study reported symptoms that are consistent with CO poisoning. This is likely an underestimate due to lack of awareness or misperception of symptoms associated with CO poisoning. Some individuals with CO poisoning symptoms may not relate lightheadedness and dizziness to CO poisoning and therefore not present to EDs. Additionally, some CO symptoms could have been considered intended effects of hookah smoking. A previous study among college students found that a commonly reported reason for hookah smoking is relaxation; hookah smokers associated feelings of relaxation with lightheadedness, dizziness, loss of balance, or a so-called “buzz” (Sharma et al. 2014).

Considerably less is reported or known about burn injuries from hookah-smoking in the existing literature. About 40% of the AIs in this study were burn injuries, most of which were due to direct contact with lit charcoals, which may also be an underestimate due to common self-treatment of superficial burn injuries. Previously, one case study related to hookah smoking AIs reported a 3-year-old child at a hookah-serving restaurant who accidentally knocked over a hookah with lit charcoal and suffered burn injuries to the hands and face (Maarschalk et al. 2017). In the case narratives, we found that some narratives reported sustained superficial and second-degree burns in a similar manner: by pulling on the hose and toppling the apparatus or otherwise accidentally knocking down the hookah with lit charcoal.

We found that two-thirds of hookah-related AIs occurred in young adults aged 18–24 years. This finding is consistent with the higher prevalence of current (past 30-day) hookah smoking in this age group according to studies using Population Assessment on Tobacco and Health (PATH) Study data (Kasza et al. 2017; Robinson et al. 2018). Another study suggests similar hookah use prevalence in adults aged 18–24 and 25–34 (Majeed et al. 2017). We also found that from 2011 to 2019, there was an overall increase in the national estimate of hookah-related AIs presenting to EDs. Youth and adult hookah use prevalence rates show fluctuations overtime throughout the study period (Kasza et al. 2018; Gentzke et al. 2019; Agaku et al. 2014). This increase in the national estimate of hookah-related AIs over time could, in part, be attributed to increasing awareness of hookah-related injuries as a result of increased hookah tobacco regulatory research and health education (Department of Health and Human Services n.d.; US Food and Drug Administration HHS n.d.). Additionally, in 2016, the US Food and Drug Administration asserted regulatory jurisdiction of all tobacco products, including hookah tobacco products through the Deeming Rule, which may have also contributed to increased awareness (US Food and Drug Administration HHS 2016).

Lastly, we found that of the AIs that presented to US EDs during the study period, more acute injuries occurred in females than in males. However, the prevalence of hookah smoking is higher among males compared to females (Robinson et al. 2018; Majeed et al. 2017). The differences between AIs and prevalence may be because women have been shown to be more likely than men to seek health care services (Bertakis et al. 2000; Thompson et al. 2016).

Our study has some limitations. One limitation is that a small number of nationally estimated cases (i.e., < 1200) is considered by CPSC to be unstable and potentially unreliable (US Consumer Product Safety Commission 2017b). Although the national estimate of the total number of cases during the study period is not subject to this limitation, our analyses that stratified by case characteristics may be unstable. Another limitation is that the lack of designated product codes for hookah tobacco in NEISS may have led to an underestimation of the number of cases; we relied on conducting a text search to identify hookah-related AI cases and may have inadvertently missed some cases. Nonetheless, this study provides the first national estimates of ED visits due to hookah-related AIs and information on AI characteristics.

Many hookah smokers may not be aware of the potential for acute injury. Because there is little known about the types of acute injuries that are sustained by using hookah, these findings could inform the development of policies to reduce the harms associated with hookah smoking. Additionally, public health educators could use these findings to increase awareness about the acute injuries associated with hookah smoking. The study findings may inform public health policy and educational intervention efforts to prevent these events and also complement other AI surveillance systems, such as the NPDS.

## Data Availability

The datasets analyzed during the current study are available in the Consumer Product Safety Commission’s National Electronic Injury Surveillance System repository, https://www.cpsc.gov/Research%2D%2DStatistics/NEISS-Injury-Data.

## Abbreviations

AI: Acute Injury
CO: Carbon Monoxide
COPD: Chronic obstructive pulmonary disease
CPSC: Consumer Product Safety Commission
FDA: Food and Drug Administration
ED: Emergency Department
NEISS: National Electronic Injury Surveillance System
NPDS: National Poison Data System
PCC: Poison Control Center
US: United States
